# Neuroendocrine tumor in the liver of a patient with isolated polycystic liver disease: A case report and review of the literature

**DOI:** 10.3892/ol.2013.1233

**Published:** 2013-03-06

**Authors:** KONSTANTINOS KOUTSAMPASOPOULOS, ELISAVET ANTONIADOU, STAVROS ZOUTIS, GRIGORIOS IACOVIDIS, OLGA BUROVA, ANTONIOS TAPLIDIS

**Affiliations:** 1 Department of Internal Medicine, General Hospital of Naoussa, Thessaloniki, Greece; 2 Second Department of Pediatrics, Aristotle University of Thessaloniki, ‘AHEPA’ Hospital, Thessaloniki, Greece; 3 Department of Radiology, General Hospital of Naoussa, Thessaloniki, Greece

**Keywords:** neuroendocrine tumor, polycystic liver disease

## Abstract

Neuroendocrine tumors (NETs) frequently metastasize to the liver, but it is rare to find them there as primary tumors. Isolated polycystic liver disease (PCLD) is a rare autosomal dominant disease. There is no known association between polycystic liver disease and neuroendocrine or other tumors. We report a case of a 64-year-old female with a past medical history of isolated PCLD who presented with increasing abdominal pain over a two-week period. Our patient underwent open surgical biopsy one month after presentation. The histological examination and immunohistochemical findings suggested an intermediate grade neuroendocrine tumor. A 24-h delayed whole-body scintigraphy technique was utilized for the identification and localization of neuroendocrine tumors via the administration of In-111-labeled OctreoScan; however, no extrahepatic accumulation was observed. No previous studies in the literature describe a patient with PCLD and a primary or metastatic neuroendocrine tumor of the liver.

## Introduction

Neuroendocrine tumors (NETs) frequently metastasize to the liver, but it is rare to find them there as primary tumors. NETs represent 2% of all tumors in the gastrointestinal tract and they have an annual incidence of 2–3 individuals out of 100,000 per year, with an overall slight preponderance in females (1). The most common primary sites are the lung, stomach, small intestine, pancreas, meckel, appendix, colon, rectum, breast, prostate, ovary, ileum, duodenum and the jejunum (1,2).

Isolated polycystic liver disease (PCLD) is a rare autosomal dominant disease with an incidence of <0.01%. The majority (>80%) of PCLD patients are clinically asymptomatic. Certain patients develop symptoms of a cystic enlarging liver, which causes morbidity and mortality (3). In PLCD, the cysts arise from malformation of the embryonic ductal plate, with formation of von Meyenburg complexes (hamartomas) that are lined with functional biliary epithelium (4). There is no known association between PCLD and neuroendocrine or other tumors.

The study was approved by the ethics committee of General Hospital of Naoussa, Naoussa, Greece. Written informed consent was obtained from the patient’s family.

## Case report

A 64-year-old female presented with increasing abdominal pain over a two-week period. The patient was a non-smoker, did not consume alcohol and denied any systemic symptoms of fever, weight loss or anorexia. No cardiac, neurological or other symptoms were reported. The patient had a past medical history of isolated PCLD, which was discovered two years previously, and an ectopic left kidney. No family history of polycystic kidney or liver disease was reported. A clinical examination revealed massive hepatomegaly, and this was confirmed by ultrasonography. No other abnormalities were detected.

Laboratory investigations revealed that the patient had normal renal function with an estimated glomerular filtration rate of >60 ml/min. The hemoglobin, white cell count differentials and coagulation screen were all within the normal range. Liver function tests revealed raised alkaline phosphatase and γ-glutamyltransferase levels of level of 475 and 531 IU/l, respectively, and a slightly low albumin level of 2.2 g/dl with a total protein level of 6.4 g/dl. The levels of total bilirubin and alanine transaminase were 2.5 mg/dl and 50 IU/l, respectively. Tumor marker levels, including those of AFP, CEA, CA 19-9 and CA 125, were not observed to be elevated.

**Figure 1:**
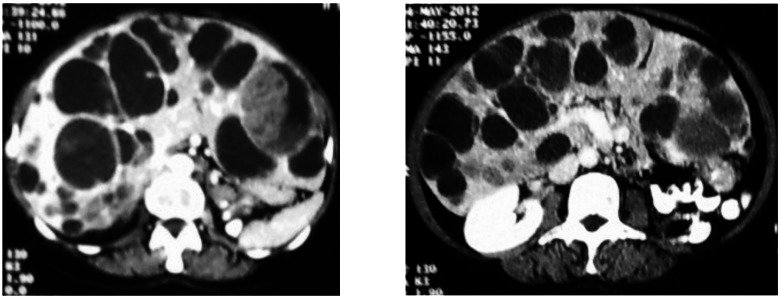
Contrast computed tomography (CT) scan reveals massive hepatomegaly and a polycystic liver with multiple cystic formations.

Contrast computed tomography (CT) examination of the abdomen confirmed massive hepatomegaly and a polycystic liver with multiple scattered cystic formations. A number of these included septals, hemorrhagic material and several nodular-compact internal lesions, which was corroborated following intravenous contrast injection (Fig. 1).

The patient underwent open surgical biopsy one month following presentation. The histological examination and immunohistochemical findings suggested an intermediate grade neuroendocrine tumor (mitotic index ki-67, 10–15%; positive for keratin 8/18, CD 56 and synaptophysin).

A 24-h delayed whole-body scintigraphy technique for the identification and localization of neuroendocrine tumors via the administration of In-111-labeled OctreoScan was used; however, no extrahepatic accumulation was observed.

Two weeks after the biopsy, the patient presented with dyspnea. A large right pleural effusion was confirmed by X-ray of the chest and triplex, while ultrasound examination of the legs revealed deep venous thrombosis (DVT). The patient succumbed a few days later.

## Discussion

Despite significant physical examination and radiological findings, the majority of patients remain asymptomatic with preserved liver function, and only mild elevations in the γ-glutamyltransferase and alkaline phosphatase levels are observed (4). Certain patients with PLCD will develop symptoms including abdominal pain, distension or early satiety due to massive hepatomegaly. More rarely, complications of cysts may include cyst infection, hemorrhage or rupture with hemoperitoneum, cyst torsion, portal hypertension, hepatic vein compression or jaundice due to bile duct compression (5,6).

Surgery remains the mainstay of treatment when patients become symptomatic. Surgical intervention comprises cyst aspiration and sclerosis, fenestration with and without hepatic resection and orthotopic liver transplantation (7).

Primary neuroendocrine tumors of the liver are rare. The frequency of primary NETs reported to occur in the liver or biliary tract is <1%. Unknown primary sites or uncommon sites account for 11–14% of cases (8). Neuroendocrine tumors originate from neuroendocrine cells from the embryological neural crest. In the gastrointestinal system, neuroendocrine cells are located from the mouth to the anus, including in the pancreas (9). Proposed theories on the origin of neuroendocrine cells that give rise to primary hepatic neuroendocrine tumors include ectopic neuroendocrine cells of pancreatic or adrenal origin, and neuroendocrine cells from within the intrahepatic biliary tree or from neuroendocrine-programmed ectoblasts (10).

There is no known association between PCLD and primary or metastatic neuroendocrine tumors. A rare correlation between PCLD and intracranial meningiomas was recently described, which was likely to have occurred by chance rather than to represent a previously unrecognized association between PCLD and cranial meningioma (11).

Additionally, a case of liver failure in a patient affected by PCLD and liver metastases from breast carcinoma was described (12). Extrahepatic abnormalities in PCLD have been described, such as mitral valve prolapse and intracranial aneurysms, but there is no known connection between PCLD and cancer (13).

Patients with neuroendocrine tumors may be evaluated by computed tomography (CT) or magnetic resonance imaging (MRI), and the functional status of these tumors is assessed by physiological imaging via scintigraphy, with agents such as radiolabeled meta-iodobenzylguanidine (MIBG) or radiolabeled octreotide (Octreoscan) (14). An-111-labeled OctreoScan is highly sensitive for the detection of gastroenteropancreatic neuroendocrine tumors and their metastases, and provides important aspects in the evaluation of these tumors for patient management (1).

No studies in the literature describe a patient with autosomal dominant isolated PCLD and a primary or metastatic neuroendocrine tumor of the liver. The death of our patient did not allow for further diagnostic examination, such as a positron emission tomography (PET) scan, to exclude any primary site other than the liver. However, the scintigraphy test and the MRI of the chest and abdomen did not reveal any suspicious regions in organs other than the liver.
